# Supplementary material for: The dynamics of overlayer formation on catalyst nanoparticles and strong metal-support interaction
Source: Nat Commun. 2020 Jun 26;11:3220. doi: 10.1038/s41467-020-17070-2 (PMC7320156; doi:10.1038/s41467-020-17070-2)
Supplement: Supplementary file 1 — Supplementary Information [file 41467_2020_17070_MOESM1_ESM.pdf]

Supplementary information

for

The dynamics of overlayer formation on catalyst nanoparticles and strong metal-support interaction

Beck et al.

Supplementary discussion

Details of the DFT results. In order to gain atomic understanding of the nature of the overlayers on the platinum nanoparticles, the effect of the gaseous environment on the relative stability of the structure of the overlayers was investigated by ab-initio atomistic thermodynamics modeling[1]. Two cases determine the thermodynamic stability of the overlayers, which depends on how many titanium atoms are reduced. In the first case, unlimited amounts of reduced titanium atoms formed from the support oxide (TiO_2). Since the surface area of platinum nanoparticles is limited, it would, therefore, be expected that the most stable overlayer is one with the lowest Gibbs free energy per surface area. In the second case, only a finite number of metal titanium atoms formed from the support oxide because of the kinetic barrier. In this case, the most stable overlayer structure is the one with lowest Gibbs free energy per reduced titanium atoms. Experiment results are supposed to fall within the range of these two cases. In 1 bar hydrogen atmosphere at 600 °C, all the reduced overlayers have negative formation energies. Thus, encapsulation of platinum nanoparticles is thermodynamically favorable under these conditions. The stability of the models containing reduced titania increases with the hydrogen pressure. The slopes of the Gibbs free energy are different for the models because the overlayers have different titanium atoms density per area (case 1) or different stoichiometry (case 2). The surface alloy of two Pt_8Ti monolayers on top of platinum is the most stable overlayer when case 2 is assumed (Figure 4 and supplementary figure 9). A mixture of reduced Ti_2O_3 and Pt_8Ti alloy is the most stable overlayer, assuming case 1, but very close in energy to the alloy and reduced titania coverage. Under 1 bar oxygen at 600 °C, all the reduced overlayers become thermodynamically unstable and re-oxidation to TiO_2 overlayers is favorable. Energy of TiO_2 overlayers on top of platinum is slightly higher (0.1 eV in case 1) compared to the TiO_2 bulk

phase; thus no significant thermodynamic driving force exists to drive the migration of oxidized TiO_2 overlayers from the platinum surface to the titania bulk. For the atomic structure of the reduced TiO_x/Pt interface, both TiO and Ti_2O_3 overlayers tend to bond with the platinum surface by titanium atoms, resulting in an arrangement exhibiting a Pt-Ti-O stacking sequence. It was assumed, therefore, that the strong bond between titanium and platinum is the main source of SMSI in the Pt-TiO_2 system.

onsiderations on the electron beam influence. The authors are aware that electron beam irradiation during in situ TEM studies can create beam-induced artifacts[2]. To minimize the likelihood of irradiation damage during the in situ experiments great care was taken to minimize the beam dose rate and exposure time. The electron beam was blanked whenever possible. The imaging conditions were kept at a beam dose rate and current density range that was previously reported for a comparable catalytic system to not alter the sample (Supplementary table 2)[3,4]. During the experiment, some areas of the sample that had not previously been exposed to the electron beam were exposed for a short period of time to obtain reference images (Supplementary figure 5). No structural differences were observed. Furthermore, to probe the influence of the beam, the experiment was repeated with extremely reduced dose rate, where the image quality was sacrificed. No change was found compared to a higher rate of dosage. The beam dose was calculated according to the following equation:

Beam dose rate = $\#_{\text{electrons per pixel}} * A_{\text{pixel area camera}} / (A_{\text{recorded image area}} * t_{\text{exposure time}})$ (1)

with $\#_{\text{electron per pixel}}$ is the averaged electron counts per camera pixel, $A_{\text{pixel area camera}}$ is the camera pixel area (2048 x 2048), $A_{\text{recorded image}}$ is the actual image size in real space, and t_{exposure} is the exposure time per image.

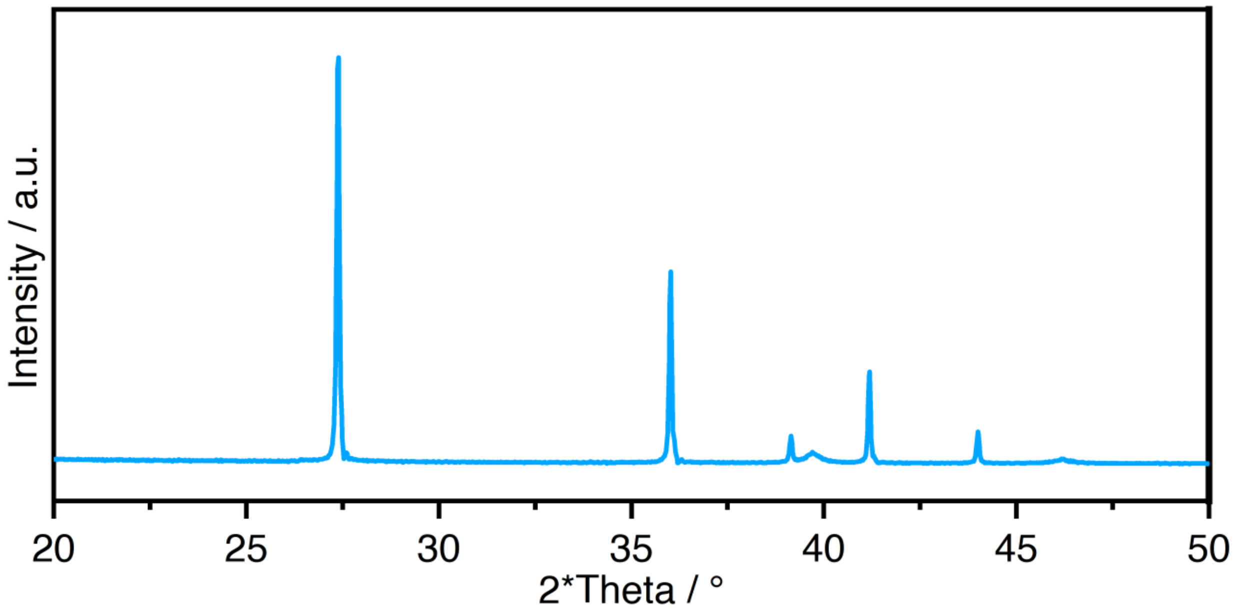

Supplementary figure 1 | X-ray diffractogram of the as prepared Pt-TiO_2 catalyst.

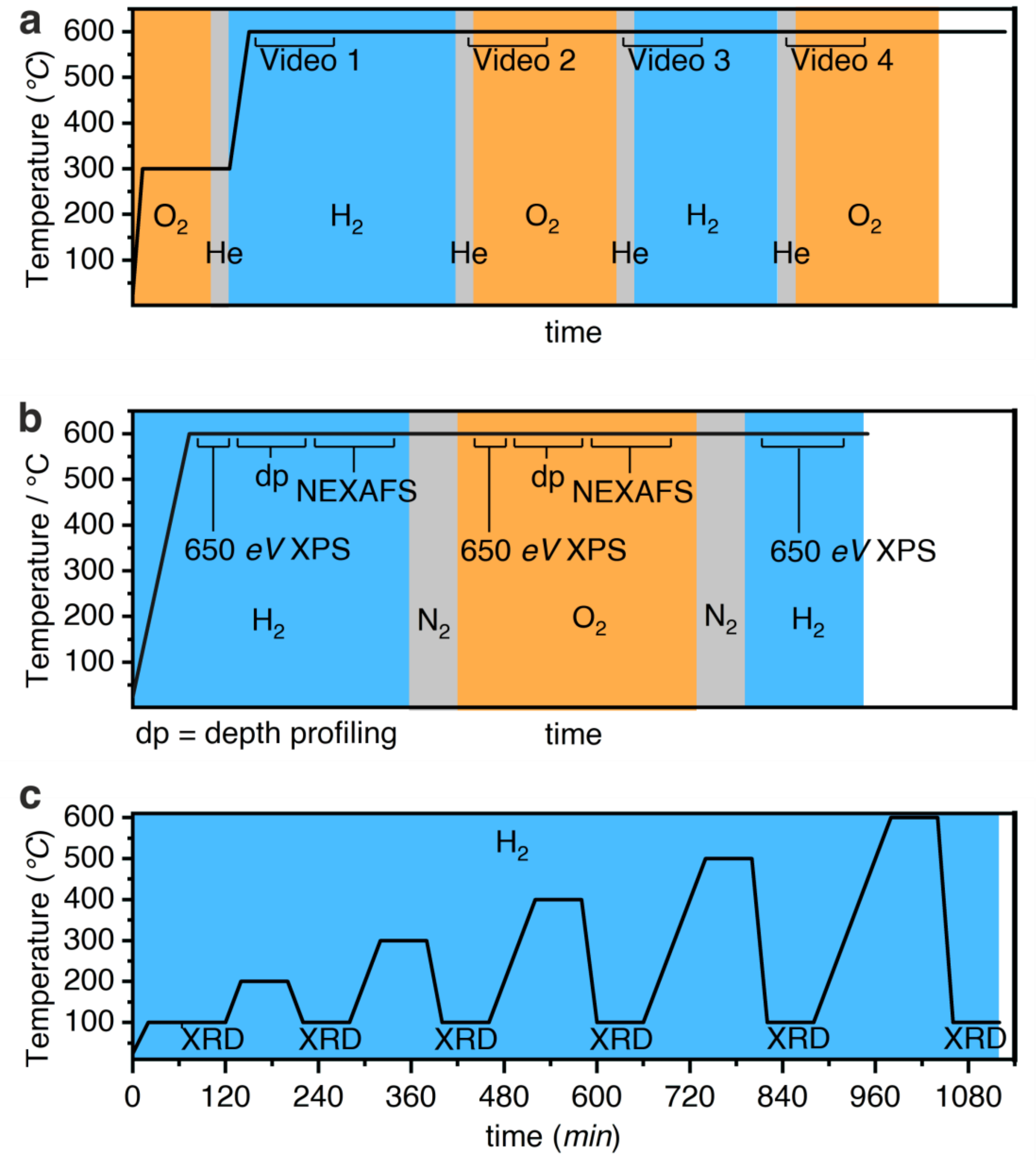

Supplementary figure 2 | a, Schematic of the performed in situ TEM experiments. **b**, Schematic of the in situ X-ray photoelectron emission experiment. Pressure at 600 °C in H_2 was 0.14 mbar and for N_2 and O_2 1.0 mbar. **c**, Schematic of the in situ powder X-ray diffraction experiment. The gas flow was kept constant at 50 ml min^{-1}; heating was performed at a heating rate of 10 °C min^{-1}.

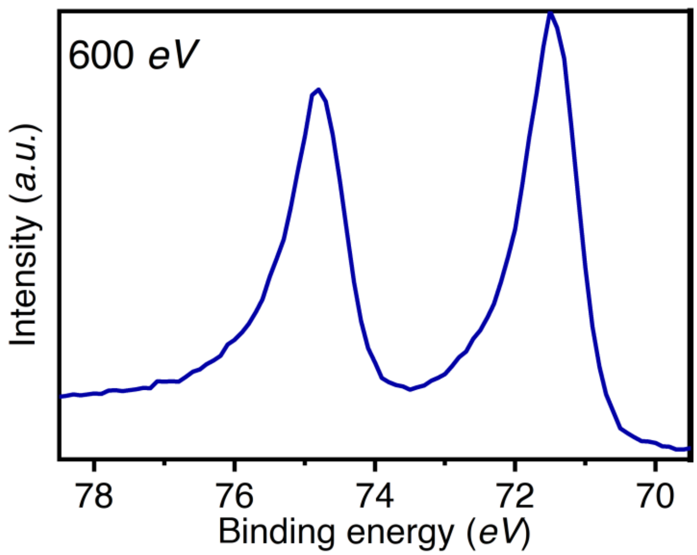

Supplementary figure 3 | Pt *4f* peak taken from a platinum foil, used as reference for Pt^0 asymmetry fitting parameters.

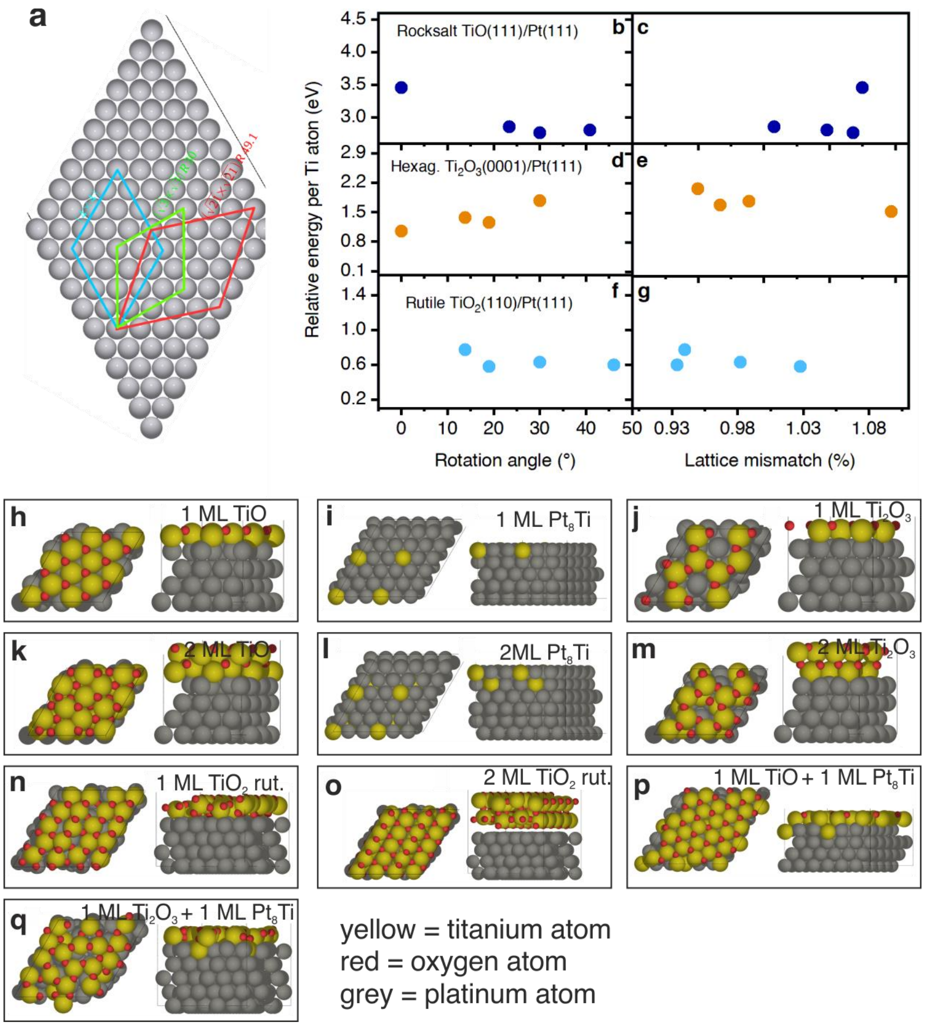

Supplementary figure 4 | a, An example of root surfaces of Pt (111). Relative energy for the rock salt TiO (111)/Pt(111) interface models as a function of (**b**) relative orientation and (**c**) lattice mismatch. Relative energy for hexagonal Ti_2O_3 (0001)/Pt(111) interface models as a function of the (**d**) relative orientation and (**e**) lattice mismatch. Relative energy for rutile TiO_2 (110)/Pt(111) interface models as a function of (**f**) relative orientation and (**g**) lattice mismatch. **h** – **q**, All calculated structures of surface models. Different types of titanium containing 1 or 2 monolayers (ML) on top of platinum layers.

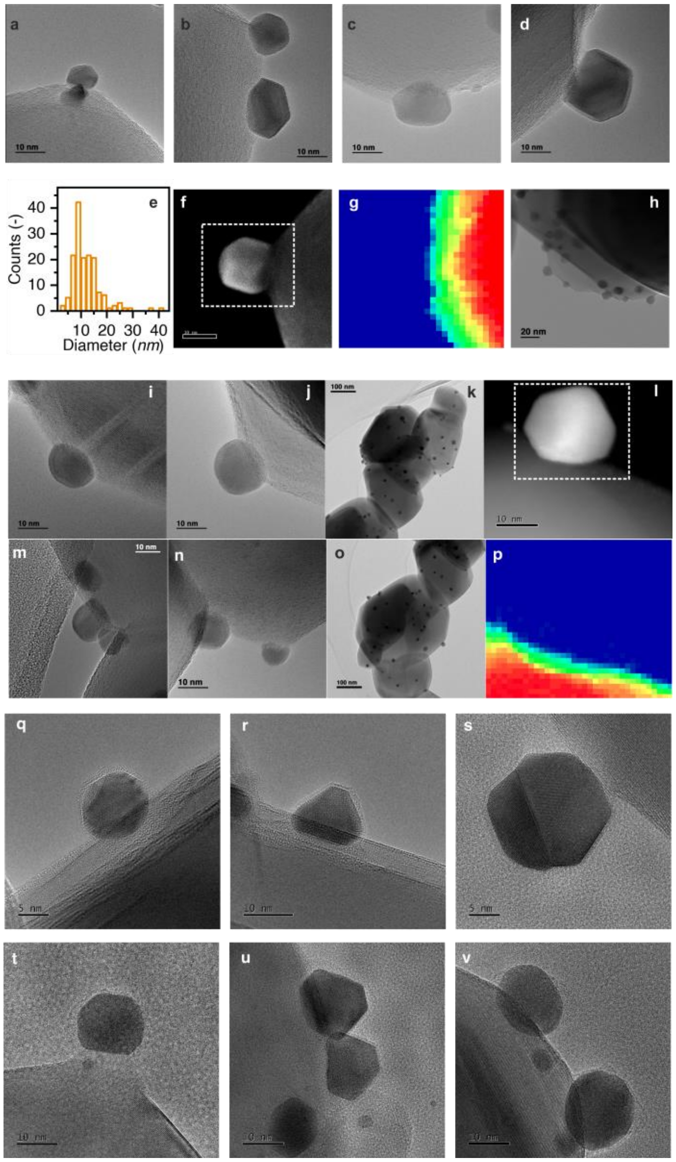

Supplementary figure 5 | a – h, Pt-TiO_2 sample as prepared. **a – d, h,** high-resolution electron micrographs. **e**, particle size distribution (N > 150). **f** secondary electron STEM image, the white square indicates the region used for EELS mapping (**g**). **i – p**, Pt-TiO_2 sample after O_2 treatment for 1 h at 600 °C. **i-j** and **l-n**: high-resolution electron micrographs. **l,** STEM image: The white rectangle indicates the region used for EELS mapping (**o**). **p-v**, In situ transmission electron micrographs at 600 °C in 1 bar of O_2. Areas were not exposed to the electron beam prior to this image acquisition.

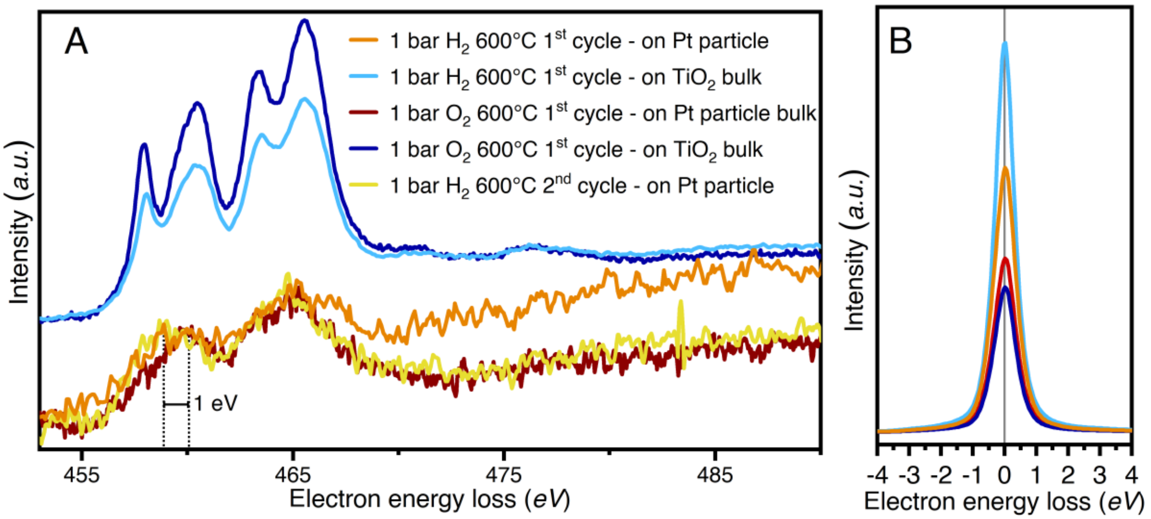

Supplementary figure 6 | Spectroscopic assessment of Electron energy loss (**A**) and alignment of the zero-loss spectra (**B**). A shows the electron energy loss spectra captured by STEM mapping in different states from different regions of the catalyst (Figure 2). The spectra of the bulk titania particles is similar under all gas conditions (red and blue curves). Thus, the titania is not reduced under the high-temperature hydrogen conditions. The much weaker Ti L-edge spectra of the platinum particles shows a shift in the edge around 458 eV of around 1 eV towards lower energy when the catalyst is in a hydrogen atmosphere compared to oxygen. This is indicative of a reduction of titanium on top of the platinum particle [5].

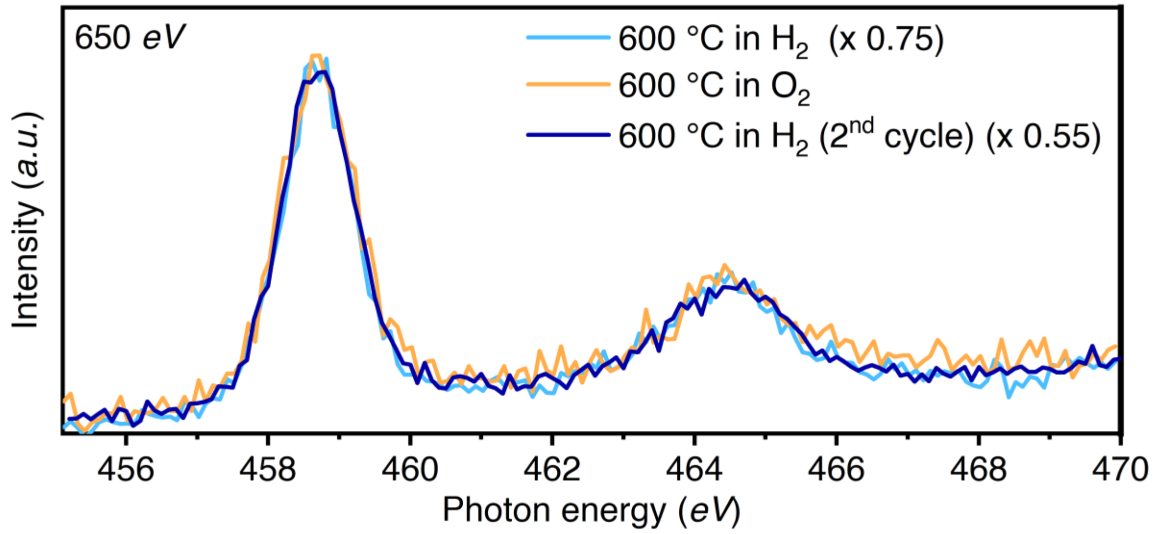

Supplementary figure 7 | Ti *2p* peak at 600 °C under H_2 (0.14 mbar) and O_2 (1 mbar) gas atmosphere. No reduction of Ti(IV) is visible. Recorded at 650 eV.

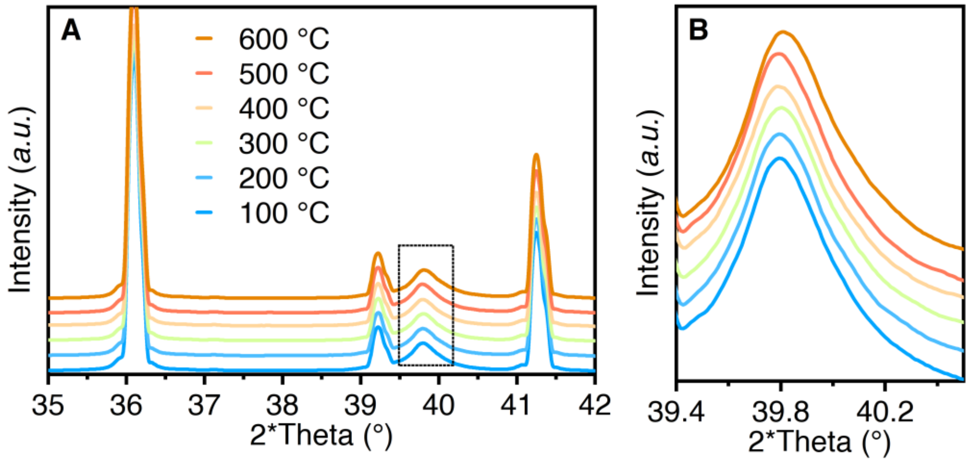

Supplementary figure 8 | In situ diffraction data of the platinum-titania catalyst in a flow of hydrogen (1 bar, 50 ml min^{-1}) recorded at 100 °C after reduction at the indicated temperature: large range (**A**) and enlarged inset of the platinum reflection region (**B**).

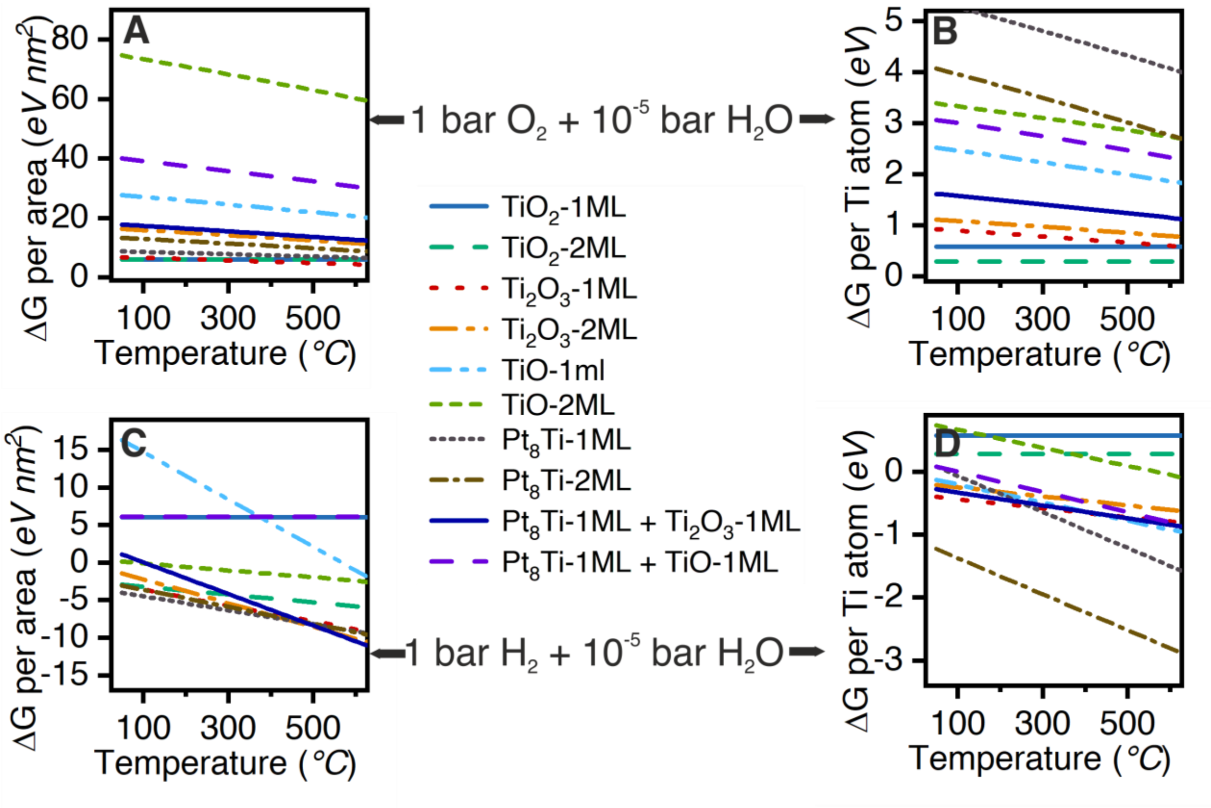

Supplementary figure 9 | Temperature dependence of the surface layer models under oxidizing (A, B) and reducing (C, D) conditions.

Kinetic photo electron energy / eV	IMFP in TiO_2 / nm	IMFP in Pt / nm	Photon energy for Pt *4f* / eV	Photon energy for Ti *2p* / eV
330	0.971	0.636	400	788
480	1.243	0.796	550	938
630	1.504	0.949	700	1088
780	1.757	1.097	850	1238
930	2.004	1.241	1000	1388

Supplementary table 1 | XPS measurement conditions and respective inelastic mean free path values for the in situ APXPS experiment.

Movie	Content	Acquisition rate / fps	Beam dose rate/ $e^- nm^{-2} s^{-1}$	Current density / A cm^{-2}
1	First exposure to H_2 at 1 bar at 600 °C	3.18	$7.3 * 10^5$	11.7
2	Gas switch from He to O_2 after the first treatment in H_2	3.18	$1.5 * 10^5$	2.2
3	Gas switch from He to H_2 after the treatment in O_2	6.27	$7.4 * 10^5$	11.8
4	Gas switch from He to O_2 after the second treatment in O_2	6.27	$1.3 * 10^6$	21.5

Supplementary table 2 | Exemplary beam dose rates and current density during the reported movies.

Supplementary references

1. Reuter, K. & Scheffler, M. First-Principles Atomistic Thermodynamics for Oxidation Catalysis: Surface Phase Diagrams and Catalytically Interesting Regions. *Phys. Rev. Lett.* **90**, 046103 (2003).

2. Sarkar, R., Rentenberger, C. & Rajagopalan, J. Electron Beam Induced Artifacts During in situ TEM Deformation of Nanostructured Metals. *Sci. Rep.* **5**, 16345 (2015).

3. Kuwauchi, Y., Yoshida, H., Akita, T., Haruta, M. & Takeda, S. Intrinsic catalytic structure of gold nanoparticles supported on TiO 2. *Angew. Chemie - Int. Ed.* **51**, 7729–7733 (2012).

4. Saavedra, J., Powell, C., Panthi, B., Pursell, C. J. & Chandler, B. D. CO oxidation over Au/TiO2 catalyst: Pretreatment effects, catalyst deactivation, and carbonates production. *J. Catal.* **307**, 37–47 (2013).

5. Zhang, Y. *et al.* Atomic Layer Deposition of Titanium Oxide on Single-Layer Graphene: An Atomic-Scale Study toward Understanding Nucleation and Growth. *Chem. Mater.* **29**, 2232–2238 (2017).
