# Supplementary material for: The dynamics of overlayer formation on catalyst nanoparticles and strong metal-support interaction
Source: Nat Commun. 2020 Jun 26;11:3220. doi: 10.1038/s41467-020-17070-2 (PMC7320156; doi:10.1038/s41467-020-17070-2)
Supplement: Supplementary file 3 — Description of Additional Supplementary Files [file 41467_2020_17070_MOESM3_ESM.pdf]

Description of Additional Supplementary Files

File Name: Supplementary Movie 1
Description: In situ TEM of the first exposure to H_2 at 1 bar at 600 °C

File Name: Supplementary Movie 2
Description: In situ TEM of gas switch from He to O_2 after the first treatment in H_2

File Name: Supplementary Movie 3
Description: In situ TEM of gas switch from He to H2 after the treatment in O_2

File Name: Supplementary Movie 4
Description: In situ TEM of gas switch from He to O_2 after the second treatment in O_2
